# Wirtschaftsprogramme gegen die Pandemiekrise — Deutschland im internationalen Vergleich

**DOI:** 10.1007/s10273-021-2917-2

**Published:** 2021-05-17

**Authors:** Michael Dauderstädt

**Affiliations:** Bonn, Deutschland

**Keywords:** O57, E58, E62

## Abstract

Die Wirtschaftsprogramme der 23 OECD-Länder setzen unterschiedliche Schwerpunkte. Im Mittelpunkt der Programme stehen Geld- und Fiskalpolitik, Einkommensunterstützung und Konjunkturprogramme, Arbeitsmarktpolitik und andere staatliche Programme in Bereichen wie Infrastruktur, Gesundheit, Bildung und Klimaschutz. Im Vergleich scheint Deutschland beim Schutz von Beschäftigung und Einkommen gut aufgestellt zu sein, wenn auch ohne einen deutlichen Fokus auf die Armutsbekämpfung. Allerdings fehlt eine klare Vision zur Ausgestaltung der Post-Corona-Ökonomie.

## Abstract (en)

This paper compares the economic programmes of 23 OECD countries with that of Germany. It focuses on monetary and fiscal policies, income support and stimulus, labour market policies and other government programmes in areas such as infrastructure, health, education and climate protection. In comparison, Germany looks well placed in protecting employment and income, albeit without a clear anti-poverty focus. However, it lacks a clear vision of the kind of post-Corona economy it wants to achieve.

Beim Vergleich der Wirtschaftspolitik zur Bekämpfung der Corona-Krise der OECD-Länder fällt auf, dass die wichtigsten staatlichen Konjunkturimpulse in den Politikfeldern Geld- und Fiskalpolitik sowie durch Hilfsmaßnahmen für Haushalte und Unternehmen, weitere Ausgabenprogramme und die Arbeitsmarktpolitik erfolgten. Seit dem Frühjahr 2020 haben die meisten Zentralbanken die Geldpolitik gelockert, um die Konjunktur anzukurbeln und die Kreditversorgung der Staaten und Unternehmen zu erleichtern. Ein weiterer, wohl auch nicht unerwünschter Nebeneffekt war die mögliche Abwertung der nationalen Währung, um die Exporte zu fördern und die Importkonkurrenz zu schwächen. Lockerung der Geldpolitik hieß in der Regel Senkung der Zinsen bzw. Beibehaltung schon vorher niedriger, manchmal sogar schon negativer Zinsen. Da die Pandemie in einer Phase bereits historisch niedriger Zinsen begann, war dieses Instrument nur begrenzt einsetzbar (Senkung um weniger als 2 Prozentpunkte). Japan und die Eurozone blieben bei den schon extrem niedrigen Zinsen (0 bzw. -0,1), wobei die Schweiz, Schweden und Dänemark de facto die EZB-Politik mit teils deutlich negativen Zinsen spiegeln. Deutschland als Teil der Eurozone genießt die gleichen geldpolitischen Impulse und profitiert mit seinen attraktiven Staatsschuldtiteln besonders vom Ankaufprogramm der EZB (vgl. Tabelle 1).

* Diese Studie wurde im Auftrag der Friedrich-Ebert-Stiftung erstellt.

**Dr. Michael Dauderstädt** ist freiberuflicher Publizist und Berater. Bis 2013 leitete er die Abteilung Wirtschafts- und Sozialpolitik der Friedrich-Ebert-Stiftung.

Angesichts der Grenzen der Zinspolitik setzten die Zentralbanken verstärkt auf das sogenannte Quantitative Easing, also den Kauf von Anleihen von Staaten und Unternehmen, um deren Liquidität, Solvenz und Kreditwürdigkeit zu stärken. Sichtbares Zeichen war die Ausdehnung der Zentralbankbilanzen, in deren Aktivseite die gekauften Vermögenstitel einflossen. Zwischen Frühjahr und Herbst 2020 nahmen viele Bilanzen um 20 % bis 40 % zu. Auch die Bilanz der Deutschen Bundesbank stieg um ca. 25 % (von unter 2 Billionen Euro im April auf knapp 2,5 Billionen Euro im November 2020). Eine Ausnahme bildeten Dänemark, Norwegen und Island. Die Anleihenkäufe der EZB machen etwa 85 % des gesamten erwarteten Haushaltsdefizits 2020/2021 der Eurozone von 1,7 Billionen Euro aus (IWF, 2020, 4).

Der unmittelbare Effekt war eine spektakuläre Erholung der Börsen, die im Sommer und Herbst 2020 wieder die Vorkrisenstände erreichten und oft überschritten. Diese Vermögenspreisinflation blieb isoliert. Eine Konsumpreissteigerung blieb weitgehend aus, aber eine deutliche Deflation konnte verhindert werden. Ein – von manchen Beobachter:innen befürchtetes – Wiederaufflammen der Inflation 2021 ist derzeit in den Markterwartungen nicht zu erkennen und wird daher von den Zentralbanken auch kaum als Risiko eingeplant. Keine Bank sieht für die nächste Zeit Zinserhöhungen vor. Die meisten Banken planen sogar noch weitere Ankäufe von Schuldtiteln. Da Staatsanleihen meist den Löwenanteil der Ankäufe ausmachen, trägt das zur weiteren Stabilisierung der Zinsbe-

Tabelle 1
**Zinspolitik ausgewählter Zentralbanken**
Zinssätze in %

| Land | Q3 2019 | Q4 2019 | Q1 2020 | Q2 2020 | Q3 2020 | Q4 2020 | Q1 2021 |
|---|---|---|---|---|---|---|---|
| Australien | 1,00 | 0,75 | 0,25 | 0,25 | 0,25 | 0,25 | 0,10 |
| Kanada | 1,75 | 1,75 | 0,25 | 0,25 | 0,25 | 0,25 | 0,25 |
| Dänemark | -0,75 | -0,75 | -0,60 | -0,60 | -0,60 | -0,60 | -0,60 |
| Eurozone | 0 | 0 | 0 | 0 | 0 | 0 | 0 |
| Großbritannien | 0,75 | 0,75 | 0,10 | 0,10 | 0,10 | 0,10 | 0,10 |
| Island | 3,50 | 3,00 | 1,75 | 1,00 | 1,00 | 1,00 | 0,75 |
| Japan | -0,10 | -0,10 | -0,10 | -0,10 | -0,10 | -0,10 | -0,10 |
| Korea | 1,50 | 1,25 | 0,75 | 0,50 | 0,50 | 0,50 | 0,50 |
| Neuseeland | 1,00 | 1,00 | 0,25 | 0,25 | 0,25 | 0,25 | 0,25 |
| Norwegen | 1,50 | 1,50 | 0,25 | 0 | 0 | 0 | 0 |
| Polen | 1,50 | 1,50 | 1,00 | 0,10 | 0,10 | 0,10 | 0,10 |
| Schweden | -0,25 | -0,25 | 0 | 0 | 0 | 0 | 0 |
| Schweiz | -0,75 | -0,75 | -0,75 | -0,75 | -0,75 | -0,75 | -0,75 |
| USA | 1,875 | 1,625 | 0,125 | 0,125 | 0,125 | 0,125 | 0,125 |

Quelle: BIS (o. D.)

lastung der Staatshaushalte bei und beugt einer Staatsschuldenpanik vor.

## Fiskalpolitik: weiter expansiv, aber schwächer

Die durch die oben beschriebene Geldpolitik „entfesselte“ Fiskalpolitik leistete den Hauptbeitrag bei der Krisenbekämpfung. Alle hier behandelten Länder haben einen – wenn auch unterschiedlich stark ausgeprägten – Wohlfahrtsstaat keynesianischer Prägung, bei dem in einer Krise zunächst die automatischen Stabilisatoren wirken, d. h. die Steuereinnahmen sinken und Ausgaben für Arbeitslosen- und Sozialhilfe steigen. In der Corona-Krise, die keine typische Rezession war, sondern sowohl angebots- wie nachfrageseitig wirkte, wurden diese automatischen Prozesse durch eine Fülle zusätzlicher Maßnahmen ergänzt und verstärkt.

Dazu zählen einnahmeseitig weitere Steuererleichterungen wie etwa auch Aufschübe bei der Zahlung von Steuern und Sozialabgaben und ausgabeseitig Zahlungen an Haushalte und Unternehmen sowie Bürgschaften und Kreditgarantien, wobei letztere den Staatshaushalt erst belasten, wenn tatsächlich der Kreditausfall eintritt. In vielen Ländern übertreffen diese Zusagen im Volumen die unmittelbaren Ausgaben, sodass der fiskalische Gesamtimpuls und die letztendliche Schuldenbelastung noch schwer abzuschätzen sind. Aber im ersten Ergebnis haben in allen Ländern gegenüber dem Vorjahr 2019 die Staatsausgaben beträchtlich zu- und die Einnahmen abgenommen.

Tabelle 2
**Haushaltssalden und Schuldenstände**
in Prozentpunkten des BIP

| Land | Veränderung Haushaltssaldo | | Veränderung Schuldenstand | |
|---|---|---|---|---|
| | 2019-2020 | 2020-2021 | 2019-2020 | 2020-2021 |
| Australien | -6,1 | -0,5 | 12,1 | 10,4 |
| Österreich | -10,3 | 3,2 | 13,4 | 3,2 |
| Belgien | -8,2 | 2,9 | 16,2 | 1,6 |
| Kanada | -11,2 | 2,9 | 9,6 | 4,0 |
| Dänemark | -7,2 | 1,7 | 4,4 | 1,0 |
| Finnland | -3,8 | 0,5 | 5,1 | 3,1 |
| Frankreich | -6,9 | 2,7 | 15,0 | 1,7 |
| Großbritannien | -11,1 | 1,7 | 18,5 | 3,4 |
| Island | -5,8 | -2,9 | 8,3 | 6,0 |
| Irland | -5,8 | -0,2 | 5,4 | 3,2 |
| Italien | -7,9 | 0,7 | 19,9 | 2,2 |
| Japan | -9,5 | 3,2 | 18,8 | 3,0 |
| Korea | -3,2 | -0,1 | 6,4 | 4,5 |
| Niederlande | -8,1 | 1,3 | 2,1 | 1,7 |
| Neuseeland | -3,4 | 0,6 | 7,7 | 7,0 |
| Norwegen | -12,5 | 6,8 | -19,3 | 11,2 |
| Polen | -7,5 | 3,5 | 12,0 | -0,3 |
| Portugal | -6,2 | 1,1 | 12,2 | 0,1 |
| Spanien | -8,6 | 2,4 | 20,1 | 2,3 |
| Schweden | -4,5 | 0,1 | 2,9 | 3,4 |
| Schweiz | -4,0 | -0,8 | 3,2 | 1,9 |
| USA | -10,2 | 0,8 | 20,2 | 5,8 |
| Deutschland | -5,7 | -1,3 | 8,6 | 2,5 |
| Durchschnitt, ohne Deutschland | -7,4 | 1,4 | 9,7 | 3,7 |

Quelle: IWF (2020); eigene Berechnungen.

Damit stiegen die Haushaltsdefizite deutlich (vgl. 2. Spalte, Tabelle 2), was auch zu einem höheren Schuldenstand (außer in Norwegen) geführt hat (vgl. 4. Spalte, Tabelle 2). Diese Werte müssen aber mit Vorsicht beurteilt werden, da sie auch einen Rückgang des Bruttoinlandsprodukts (BIP) widerspiegeln, das ja bei den gewählten Indikatoren im Nenner steht. Für das Jahr 2021 planen die meisten Länder niedrigere Haushaltsdefizite (vgl. 3. Spalte, Tabelle 2), weil sie von einer Wiederbelebung der Konjunktur ausgehen, aber auch eine weitere Zunahme der Staatsverschuldung vermeiden oder zumindest bremsen wollen. Wie man beim Vergleich von Spalte 2 und 3 der Tabelle 2 sehen kann, würde aber in keinem Land das Defizit auf das Niveau von 2019 abgesenkt werden, sondern weiter deutlich darüber liegen. Das gilt besonders für Deutschland, dessen Defizit 2021 sogar über 2020 liegen dürfte. Im Durchschnitt nahm das Defizit von 2019 auf 2020 um über 7 Prozentpunkte des BIP

Tabelle 3
**Hilfsprogramme**

| Land | Für Haushalte | Für Unternehmen | Sonstige Ausgabenprogramme |
|---|---|---|---|
| Australien | Steuererleichterungen | Günstigere Abschreibungsregelungen; Verlustrücktrag | Forschung und Entwicklung, Klimaschutz, Infrastrukturprojekte |
| Österreich | Einkommensteuersatzsenkung in der 1. Steuerklasse von 25 % auf 20 % | Steuererleichterungen und Mehrwertsteuersenkung in ausgewählten Branchen und für bestimmte Investitionen; Notfallhilfen für kleine Unternehmen; Umsatzverlustersatz (bis 80 %) | Klimaschutz, digitales Lernen |
| Belgien | Aussetzung der Bedienung von Hypothekenkrediten | Einkommensersatz für Selbständige; Kreditgarantien und Rückzahlungserleichterungen für Altkredite | |
| Kanada | Steuerstundung (läuft aus) | Hilfen für Mieten, Kredite und Kreditgarantien | |
| Dänemark | Freigabe von Rentenzahlungen (2,6 % des BIP) | Liquiditätshilfen, Kreditgarantien, gezielte Subventionen; Rekapitalisierungsfonds; Ersatz von Lohnfortzahlungen im Krankheitsfall | Sozialer Wohnungsbau, kommunale Ausgabenprogramme |
| Finnland | | Unterstützung kleine und mittlere Unternehmen und besonders betroffene Branchen (Gastronomie, Reisen) | |
| Frankreich | Hilfen für Obdachlose, Studierende; Schulkinder | Steuererleichterungen; Aussetzung von Steuer- und Sozialbeiträgen; Stundung von Mieten und Versorgungskosten; Subventionen für Autokauf; Hilfen für Automobil-, Tourismus- und Luftfahrtsektor; Kreditgarantien und Quasi-Eigenkapitalbeteiligungen | Öffentliche Investitionen (France Relance Programm); Hilfsprogramm für Start-ups |
| Großbritannien | Einkommenshilfen Universal Credit und Working Tax Credit erweitert, 8 Mrd. GBP | Kreditprogramme: The Bounce Back Loan Scheme; Coronavirus Business Interruption Loan Scheme, Covid Corporate Financing Facility, Steuerstundung (40 Mrd. GBP) | 31,9 Mrd. GBP für NHS; 4 Mrd. GBP Bauinvestitionen, Zuschüsse an Bahn (3,5 Mrd. GBP) |
| Island | Freigabe von Zugriff auf Lebensversicherungen | Hilfen für Firmen mit mehr als 75 % Umsatzeinbußen; Kreditgarantie für Icelandair | Mehr öffentliche Investitionen, vor allem digitale Infrastruktur |
| Irland | Einkommenshilfen für wegen der Pandemie Entlassene | Steuerstundung, Kreditprogramme, sonstige Maßnahmen zur Insolvenzvermeidung, Mehrwertsteuersenkung um 2 Prozentpunkte; Steuererleichterungen für Selbständige | Mehrausgaben für Gesundheit und Brexit-Folgen |
| Italien | Kreditstundungen bei Erstwohnungskäufen; Kindergeld; Steuerstundung | Steuerstundung; Sozialbeiträge ausgesetzt, Garantien für alte und neue Kredite | Öffentliches Investitionsprogramm für grünes, digitales und inklusives Wachstum |
| Japan | JPY 100.000 (= 700 Euro) Einmalzahlung für jeden Einwohner; Extrazahlungen an Alleinerziehende | Direktzahlungen an besonders betroffene Geschäfte; Mietsubventionen | Förderung des Inlandstourismus |
| Korea | Konsumhilfen für ärmere Haushalte | Stundung von Steuern und Sozialbeiträgen; Kredithilfen für kleine und mittlere Unternehmen | Korean New Deal (digital and green) |
| Niederlande | Einkommenshilfen für Selbständige (max. 1.500 Euro); Hilfen für Eltern, ohne Kinderbetreuung | Lohngarantien und Zuschüsse für Kleinunternehmen; Steuerstundung bis 2022; keine Zinsen auf Steuerschulden; zusätzliche Kreditlinien | 800 Mio Euro extra für Gesundheitssektor |
| Neuseeland | | Lohnsubventionen; Krediterleichterungen; Pauschalhilfen (4.000 Euro) für geschlossene Geschäfte; Steuerstundungen | New Zealand Upgrade Programme |
| Norwegen | Verlängerung von Einkommenshilfen | Fixkostenersatz; Rückstellungen für Kreditausfallgarantien; Hilfen für Luftfahrtbranche | Mehrausgaben für Forschung und Entwicklung |
| Polen | Höhere Sozialleistungen und Renten | Liquiditätshilfen | Überregionale Infrastruktur |
| Portugal | Lohnerhöhungen für Beschäftigte im Gesundheits- und Niedriglohnsektor; höheres Arbeitslosengeld; Hilfen für Eltern ohne Kinderbetreuung; Mietstundung | Verzögerte Zahlung von Sozialbeiträgen, Mehrwert- und Unternehmenssteuer; Kreditlinien für kleine und mittlere Unternehmen, Tourismus, Reisebüros und alle Unternehmen, die keine Entlassungen vornehmen | Digitalisierung und Klimaschutz |
| Spanien | Hilfen für schwache Mieter<br>Hilfen für Selbständige | Kreditgarantien (40 Mrd. Euro) für Investitionen; Fonds (10 Mrd. Euro) für strategische Unternehmen; Hilfen für Automobil-Tourismus und Transportbranchen; Kreditstundungen; Aussetzung von Insolvenz | Fonds für Regionalregierungen für Gesundheitskosten (2,9 Mrd. Euro) + Sondermedizinfonds (1,4 Mrd. Euro) |
| Schweden | Einkommenssteuersenkung | Senkung der Arbeitgeberbeiträge | Infrastruktur und „green economy“ |
| Schweiz | Einkommensersatzleistungen für Beschäftigte und Selbständige | Kreditgarantien und Steuerstundungen für notleidende Unternehmen | |
| USA | Einmalzahlungen 2020 (1.200 US-$ + 500 US-$/Kind) an Familien; 2021: 600 US-$; Aufstockung Medicare und food stamps; Aussetzung Bedienung Studentenkredite | (Teils nicht rückzahlbare) Kredite an kleine und mittlere Unternehmen; Steuerstundungen; Kreditlinien für notleidende Unternehmen (500 Mrd. US-$) | Zuschüsse an Bundesstaaten und Gemeinden; 117 Mrd. US-$ für Krankenhäuser und Veteranen |

Tabelle 3 (Fortsetzung)
**Hilfsprogramme**

| Land | Für Haushalte | Für Unternehmen | Sonstige Ausgabenprogramme |
|---|---|---|---|
| Deutschland | Mehrwertsteuersenkung bis Ende 2020, 7,7 Mrd. Euro Erleichterung Zugang zur Sozialhilfe (ALGII), 5,2 Mrd. Euro zusätzliche Kranken- und Pflegeversicherung, Kinderbonus 300 Euro/Kind, „Sozialgarantie 2021" (Deckelung), EEG-Umlage | Steuerstundungen, Verlustrücktrag und Abschreibungsregeln (7,3 % des BIP), Wirtschaftsstabilisierungsfonds mit Liquiditätshilfen und Kreditgarantien (24 % des BIP), Umsatzverlustersatz, 25 Mrd. Euro Neustart, 50 Mrd. Euro für KMU und Selbständige, Beteiligung bei Lufthansa und TUI | 2 Mrd. Euro für Start-ups, 6 Mrd. Euro E-Mobilität, 4,9 Mrd. Kommunen, 4,4 Mrd. Euro Künstliche Intelligenz, 4 Mrd. Gesundheitssystem, 4 Mrd. Euro Öffentliche Investitionen, 3,6 Mrd. Euro Wasserstoff-Strategie, 2,1 Mrd. Digitalisierung Verwaltung und mehr |

Quellen: OECD (2020); Bruegel (2020); ILO (2020); diverse nationale Quellen.

zu (Deutschland etwas unterdurchschnittlich). Für 2021 erwartet der Internationale Währungsfonds (IWF) im Durchschnitt ein um 1,4 Prozentpunkte niedrigeres Defizit, das aber immer noch 6 Prozentpunkte über dem Niveau von 2019 liegen würde. Insofern bleibt die Fiskalpolitik weiter expansiv. Auch die Verschuldung stieg gegenüber 2019 durchschnittlich um knapp 10 Prozentpunkte an, wobei Deutschland mit 8,6 % etwas darunter liegt. Auch 2021 wird die Verschuldung in allen Ländern voraussichtlich weiter ansteigen (vgl. 4. Spalte, Tabelle 2). Letztlich machen die Länder eine Abkehr von der stark expansiven Fiskalpolitik von der weiteren Entwicklung der Pandemie und der Konjunktur abhängig. Die derzeitigen Prognosen hoffen auf eine Erholung in der zweiten Jahreshälfte 2021, um dann die diversen Unterstützungsprogramme zurückfahren zu können.

## Hilfsprogramme

Die staatlich verordneten Einschränkungen trafen und treffen viele Haushalte und Unternehmen hart. Um deren Einkommensverluste zu kompensieren und dadurch die Konsumnachfrage der Haushalte und das Überleben der betroffenen Unternehmen zu sichern, legten die Regierungen zahlreiche Hilfsprogramme auf (vgl. Tabelle 3). Sie umfassten direkte Zahlungen, Erleichterungen bei Steuern und Sozialabgaben, Kredite und Bürgschaften sowie gelegentlich auch Unternehmensbeteiligungen. Zu diesen Hilfsprogrammen zählen auch die arbeitsmarktpolitischen Maßnahmen (vor allem Kurzarbeitsregelungen und die konventionelle Arbeitslosenversicherung), auf die gesondert eingegangen wird.

In der Summe sind diese Programme hauptsächlich für die steigenden Haushaltsdefizite (vgl. Tabelle 2) verantwortlich, da sie höhere Ausgaben und niedrigere Einnahmen (wegen der Steuererleichterungen) verursachen. In ihrer Masse sind sie strukturkonservativ, da sie versuchen, bestehende Einkommensstrukturen zu erhalten. Zukunftsweisende Maßnahmen finden sich in Spalte 3 der Tabelle 3 und konzentrieren sich auf Klimaschutz, Digitalisierung, E-Mobilität, Infrastruktur, Forschung und Entwicklung. Nicht berücksichtigt sind hier die geplanten Maßnahmen mit Mitteln aus dem im Juli 2020 beschlossenen EU-Programm Next Generation EU, da sie erst im Laufe von 2021 zum Einsatz kommen.

Eine Sonderstellung nehmen die Ausgaben ein, die direkt zur Bekämpfung der Pandemie dienen (z. B. für Krankenhäuser oder Impfstoffe). Zwar sind generell die Ausgaben für Gesundheit während der Pandemie nicht gestiegen (EIU, 2020), da andere Behandlungen unterlassen wurden, aber es kommen noch erhebliche Ausgaben für Impfungen auf die Länder zu. Die Kosten für den Impfstoff allein können bis zu 70 Euro/Impfling betragen. Sollten nur 60 % der Bevölkerung geimpft (um Herdenimmunität zu erreichen) werden, so liegt der Aufwand bei ca. 40 Euro/Einwohner. Für die USA sind das etwa 13 Mrd. Euro, für Deutschland über 3 Mrd. Euro, für große europäische Länder über 2 Mrd. Euro, für kleinere immer noch 400 Mio. Euro. Dazu kommen noch die Kosten der Impfaktion selbst.

## Arbeitsmarktpolitik

Ein starker Anstieg der Arbeitslosigkeit war unvermeidlich angesichts des staatlich verordneten Lockdowns und der Zurückhaltung der Bevölkerung, bestimmte, kontaktintensive Dienstleistungen in Anspruch zu nehmen. Die Reaktion der Regierungen lässt sich in zwei Varianten unterteilen: Beschäftigungsstabilisierung, vor allem durch Kurzarbeit, und Verbesserung der traditionellen Arbeitslosenunterstützung. Bei der Beschäftigungsstabilisierung befürchten manche Fachleute eine zu starke Strukturkonservierung, die eine Reallokation der Arbeit behindert. Das einzige Land ohne solche Programme sind die USA. Vor allem in der Phase des Abklingens der Krise treten dazu Maßnahmen der Beschäftigungsförderung (z. B. Lohnsubventionen bei Neueinstellungen, Ausbildung). Dazu zählen im weiteren Sinne auch viele der Programme, die die Kaufkraft stabilisieren und private und öffentliche Investitionen fördern und so dazu beitragen, neue Jobs zu schaffen.

Tabelle 4

**Maßnahmen der Arbeitsmarktpolitik**

| Land | Beschäftigungsstabilisierung | Verbesserung der Arbeitslosenunterstützung | Beschäftigungsförderung |
|---|---|---|---|
| Australien | Jobkeeper-Programm | | Jobmaker-Programm |
| Österreich | Kurzarbeit bis März 2021 | | |
| Belgien | Zeitweise Entlassung | | |
| Kanada | Lohnsubvention (CEWS) bis Mitte 2021 | Einkommenssicherung durch Canada Emergency Response Benefit (CERB) | |
| Dänemark | Jobsicherung (8 % aller Beschäftigten) nur noch begrenzt; neues Kurzarbeitsprogramm | | |
| Finnland | Zeitweise Entlassung | Zugang zur Arbeitslosenversicherung erleichtert und auf Selbständige ausgedehnt | |
| Frankreich | Verstärktes Kurzarbeitsprogramm | | Finanzierung für Ausbildung |
| Großbritannien | Jobsicherungsprogramm zahlt 80 % der Löhne und läuft bis 31.3.21 | | |
| Island | Kurzarbeitsprogramm | | |
| Irland | Lohnsubventionen erweitert auf Saisonarbeit | | |
| Italien | Kündigungsverbot | | Erleichterung von Zeitverträgen |
| Japan | Finanzierung von Sonderurlaub | | |
| Korea | Lohnsubventionen | | |
| Niederlande | Jobsicherungsprogramm bis Juli 2021; danach langsamer Ausstieg aus Lohnsubventionen | | |
| Neuseeland | Jobsicherung durch Lohnsubvention (14 Mrd. NZD = 4,5 % BIP) | | |
| Norwegen | Partieller Ersatz der Lohnfortzahlung durch staatliche Lohnsubvention | | |
| Polen | Jobsicherungsprogramm wird zurückgefahren | Mindestlohn erhöht | |
| Portugal | Jobsicherungsprogramm verlängert für 2021; Kreditlinien für alle Unternehmen, die keine Entlassungen vornehmen | Erhöhung von Löhnen und Arbeitslosengeld | |
| Spanien | Kurzarbeit verlängert bis 31.1.21; Sozialversicherungsbeiträge ausgesetzt (9,1 Mrd. Euro) | Erhöhung von Löhnen und Arbeitslosengeld | |
| Schweden | Kurzarbeitergeld | Erhöhung des Arbeitslosengelds | Trainingsangebot für Randgruppen des Arbeitsmarkts |
| Schweiz | Kurzarbeit ausgedehnt | Arbeitslosenversicherung wird durch Fonds aufgestockt (1,7 % BIP) | |
| USA | | Aufstockung der Arbeitslosenhilfe; 2021: 300 US-$/Woche bis März | |
| Deutschland | Kurzarbeitergeld (23,5 Mrd. Euro) | Erleichterung bei Hartz IV | 500 Mio. Euro Ausbildungsbeihilfen |

Quellen: OECD (2020); Bruegel (2020); ILO (2020); diverse nationale Quellen.

Tabelle 4 gibt einen Überblick über die Maßnahmen in den ausgewählten Ländern. Wie man sieht, lag der Schwerpunkt in der Beschäftigungssicherung durch partielle staatliche Übernahme der Lohnzahlungen (Ausnahme: USA). Viele dieser Programme wurden über ihren ursprünglichen Zeitrahmen über 2021 hinaus verlängert. Es bleibt abzuwarten, welche beschäftigungspolitischen Strategien langfristig erfolgreicher sind. In jedem Fall sind Maßnahmen sinnvoll, die die Qualifizierung und Umschulung von Arbeitskräften unterstützen, um ihre Integration in eine – möglicherweise stark veränderte – Post-Corona-Ökonomie zu erleichtern. Je weniger stark die Pandemie die Strukturen verändert, desto nützlicher sind Politiken, die auf den Erhalt von bestehenden Arbeitsplätzen setzen.

## Deutschland im internationalen Vergleich

Deutschland ging ins Coronajahr 2020 mit einer im internationalen Vergleich guten Wirtschaftslage: Die Beschäftigung war hoch und die Arbeitslosigkeit relativ niedrig (3 %) trotz schwachen Wachstums 2019. Der Staatshaushalt war mehr als ausgeglichen („schwarze Null“) und der

Tabelle 5
**Deutschland im internationalen Vergleich**

| Politikfeld | Deutschland | Vergleichsländer |
|---|---|---|
| Geldpolitik | EZB-Politik sorgt für Negativrendite neuer Staatschulden | Ähnliche Politik mit weniger deutlichen Effekten in schon stark verschuldeten Ländern |
| Fiskalpolitik | Hohes Defizit (auch noch 2021) und entsprechender Schuldenanstieg | Große Länder (G7 außer Frankreich) expansiver, sonst oft vorsichtiger; öfter mittelfristig steigende Schuldenstandsquoten |
| Hilfsprogramme | Einkommensstabilisierung dominiert | Einkommensstabilisierung dominiert auch in den meisten Ländern (weniger stark in Korea, Neuseeland) |
| für Haushalte | Hilfen außer Kinderbonus nur indirekt (Mehrwertsteuer, Energie, Deckelung Sozialbeiträge) regressiv mit geringer Konsumwirkung | Direktzahlungen (z. B. USA, Japan), Erleichterung bei Steuern und Sozialabgaben |
| für Unternehmen | Umfangreiche Hilfen in unterschiedlichen Formaten; werden nicht voll ausgeschöpft; eher strukturkonservativ | Ähnliche Hilfen in leicht unterschiedlichem Mix, Erleichterungen/ Stundungen von Steuern und Abgaben, Zuschüssen, Krediten etc. |
| Sonstige Programme | Diverse Investitionsprogramme; Einstieg in Digitalisierung und Klimaschutz, relativ schwach ausgelegt; Mittelabfluss langsam | Ähnliche Schwerpunkte; interessant: Wohnungsbau in Dänemark; Volumen variieren |
| Arbeitsmarktpolitik | Beschäftigungssicherung dominiert | Beschäftigungssicherung dominiert in den meisten Ländern (Ausnahme USA) |
| Beschäftigungssicherung | Kurzarbeit (wie schon 2009) erfolgreich | Viele Länder übernahmen Kurzarbeit von Deutschland |
| Arbeitslosenunterstützung | Nur relativ geringe Erleichterungen | Viele Länder haben Zahlungen an Arbeitslose verbessert (z. B. USA, Schweden, Portugal) |
| Beschäftigungsförderung | Ausbildungsbeihilfen | Schwachpunkt in vielen Ländern |

Quelle: Eigene Erhebungen.

Schuldenstand niedrig (unter 60 % des BIP). Zu Beginn der Pandemie im Frühjahr 2020 reagierte das Land etwas zögerlich, aber dann doch entschlossen, sodass in der ersten Welle relativ wenig Opfer zu beklagen waren. Der Konjunktureinbruch war trotzdem massiv. Die zweite, tödlichere Welle ab Oktober 2020 bedroht die Erholung. Die wirtschaftspolitische Reaktion brach mit der heiligen Kuh „schwarze Null" und lieferte einen für Deutschland ungewöhnlich starken, schuldenfinanzierten Konjunkturimpuls, der durch die Geldpolitik der EZB massiv erleichtert wurde. Der Mix zwischen Einkommenssicherung und Modernisierung war eher konservativ. Einen detaillierten Vergleich nach Politikfeldern liefert Tabelle 5.

Allgemein ist festzuhalten, dass sich die Konjunkturprogramme innerhalb der OECD-Länder stark ähneln. Zwar gibt es in jedem Land andere Akzente und spezielle Programme, aber der grundsätzliche Instrumentenkasten in den diversen Politikfeldern ist weitgehend derselbe, wenn auch die Einsatzintensität variiert. Deutschland liegt im Mittelfeld und damit unter seinen Möglichkeiten angesichts der guten Ausgangslage. Eine solide Bewertung der Programme würde eine Wirkungsanalyse erfordern, für die bisher kaum belastbare Daten vorliegen. Eine Kausalzuordnung von Ergebnissen (z. B. Wachstums- und Arbeitslosigkeitsraten) zu Politiken wäre zusätzlich problematisch. Man könnte immer einwenden, dass es ohne diese Maßnahmen schlimmer gewesen wäre. Im Ländervergleich müsste außerdem die unterschiedliche Wirtschaftsstruktur, politische Handlungsfähigkeit und Corona-Betroffenheit der Vergleichsländer berücksichtigt werden. Daher seien hier nur einige vorläufige Bemerkungen zum deutschen Konjunkturpaket gemacht:

- *Pluspunkte*: Pragmatische Bereitschaft zu höheren Ausgaben und Staatsschulden; großzügige Kurzarbeitsregelung (traditionelle deutsche Stärke); einige Zukunftsinvestitionsprogramme (wie Digitalisierung, E-Mobilität, Klimaschutz).
- *Minuspunkte*: Hilfen ohne gezielte direkte Förderung des Konsums (Mehrwertsteuersenkung fast nutzlos); einseitiger Fokus auf Einkommens- und Beschäftigungssicherung ohne deutliche Priorisierung der sozial Schwachen; keine klare, kohärente Zukunftsstrategie für eine Post-Corona-Wirtschaft mit entsprechenden Investitionsprioritäten.

## Ausblick und Exit-Strategie

Die meisten Länder haben angesichts der zweiten Welle und der absehbar noch länger andauernden Pandemie ihre Stützungsprogramme 2021 verlängert. Erst für das zweite Halbjahr rechnen viele Regierungen, internationale Organisationen und Forschungsinstitute mit einer Konjunkturerholung. Dann können viele Programme auslaufen, womit die Staatsausgaben auf ihr normales Niveau zurückkehren und die Einnahmen bei wieder „normaler“ Besteuerung zunehmen. Umstritten ist allerdings das Tempo der Normalisierung. Eine zu schnelle Konsolidierung der Staatshaushalte könnte die aufkommende Konjunktur abwürgen. Diese Gefahr droht in Deutschland ab 2022 (nach der Bundestagswahl). Ähnliches gilt für die Geldpolitik. Die meisten Zentralbanken planen nur ein langsames Zurückfahren der Anleihekäufe und ein noch späteres Anheben der Zinsen, solange eher Deflations- als Inflationsgefahren drohen und die hochverschuldeten Staaten bei höheren Zinsen in eine Schuldenkrise geraten könnten.

Unklar ist auch der Weg zu einem Schuldenabbau, soweit er überhaupt ein wünschenswertes Ziel darstellt. Selbst eine längere Phase ausgeglichener Haushalte würde nur bei ordentlichem nominalen BIP-Wachstum die Schuldenstandsquote (= Schulden/BIP) langsam sinken lassen. Optionen eines schnelleren Schuldenabbaus durch Haushaltsüberschüsse würden Ausgabenkürzungen und/oder Steuererhöhungen erfordern. Beides könnte das Wachstum einbrechen lassen und eine Deflation auslösen, womit – entgegen dem eigentlichen Ziel – die Schuldenstandsquote wieder steigen würde (wie z. B. in Griechenland durch die Sparpolitiken ab 2010). Dies könnte eventuell vermieden werden, wenn die Steuererhöhungen weder Konsum noch Investitionen beeinträchtigen würden, was vor allem bei einer Besteuerung hoher Einkommen und Vermögen wahrscheinlich wäre. Noch radikalere Konzepte sehen eine Vermögensabgabe (etwa nach dem Modell des deutschen Lastenausgleichs nach dem Zweiten Weltkrieg) vor.

Oft wurde gefordert, die massiven Staatsausgaben in der Pandemie dafür einzusetzen, ohnehin notwendige Modernisierungsprozesse umzusetzen oder zumindest anzustoßen („Building back better“). Diese Priorität reibt sich mit den etablierten Interessen, bestehende Einkommensquellen zu erhalten. Obendrein konkurrieren auch innerhalb dieses weit gefassten Imperativs unterschiedliche Ziele: Klimaschutz, bessere Vorbereitung auf künftige Pandemien, Digitalisierung (vor allem im Bildungs- und Gesundheitssystem), E-Mobilität, Forschung und Entwicklung und Behebung von Defiziten in der Infrastruktur. Sie alle finden sich in wechselnder Zusammensetzung in den meisten nationalen Programmen, haben aber quantitativ oft ein vergleichsweise geringes Gewicht gegenüber der Einkommenssicherung. Das gilt auch für Deutschland. Indirekt trägt aber auch die Einkommenssicherung zur Modernisierung der Wirtschaft bei, wenn etwa Haushalte die Finanzmittel für neue Bedürfnisse (z. B. Digitalisierung) einsetzen, die sie erhalten und nicht für herkömmliche Zwecke (wie Reisen, Veranstaltungen, Gastronomie etc.) ausgeben wollen oder können.
